# A radiomics model that predicts lymph node status in pancreatic cancer to guide clinical decision making: A retrospective study

**DOI:** 10.7150/jca.61101

**Published:** 2021-08-22

**Authors:** Xiaoyuan Liang, Wei Cai, Xingyu Liu, Ming Jin, Lingxiang Ruan, Sheng Yan

**Affiliations:** 1 Department of Hepatobiliary and Pancreatic Surgery, The Second Affiliated Hospital, School of Medicine, Zhejiang University, Hangzhou, Zhejiang 310009, China.; 2 Radiology Department, The First Affiliated Hospital, School of Medicine, Zhejiang University, Hangzhou, Zhejiang 310003, China.; 3 The First Affiliated Hospital of USTC, Division of Life Sciences and Medicine, University of Science and Technology of China, Hefei, Anhui, 230001, China.

**Keywords:** Pancreatic ductal adenocarcinoma, Noninvasive tool, Lymph nodes, Radiomics technology

## Abstract

**Purpose:** To construct a radiomics-based model for predicting lymph node (LN) metastasis status in pancreatic ductal adenocarcinoma (PDAC) before therapy and to evaluate its prognostic clinical value.

**Materials and Methods:** We retrospectively collected preoperative CT scans of 130 PDAC patients who underwent original tumor resection and LN dissection in the entire cohort between January 2014 and December 2017. Radiomics features were systematically extracted and analyzed from CT scans of 89 patients in the primary cohort. To construct a radiomics signature, the least absolute shrinkage and selection operator methods were employed with LN metastasis status as classification labels. Pathological analysis of LN status which were assessed by experienced pathologists was used as the evaluation label. We subjected the clinical nomogram to multivariable logistic regression analysis and conducted performance evaluation based on its discrimination, calibration, and clinical value. The model was tested and validated in 41 patients with PDAC in a separate validation cohort.

**Results:** Four radiomics features closely associated with LN metastasis were selected in the primary and validation cohorts (*P* < 0.01). Following the integration of CT-reported results and radiomics signatures into the radiomics nomogram, we reported better performance in the primary (area under the curve, 0.80) and validation (area under the curve, 0.78) cohorts.

**Conclusion:** The noninvasive tool constructed from the portal venous phase CT based on radiomics showed better performance for LN metastasis prediction than traditional approaches in pancreatic cancer. It may assist surgeons in crafting detailed procedures before treatment, this subsequently improves tumor staging and resection of patients.

## Introduction

Reports classify pancreatic ductal adenocarcinoma (PDAC) among the most lethal cancers. It is characterized by poor diagnosis and prognosis. Despite advancements in medical technology, the 5-year overall survival rate of PDAC still is as low as 6% 1, 2. Among the clinical factors, lymph node (LN) status is potentially key in assessing the stage of the disease, this consequently influences the formulation of surgical procedures 3, 4. LN status may influence treatment decisions, whereas insufficient examination of LN status results in the misclassification of PDAC patients who end up missing the best opportunity of surgery or neoadjuvant therapy. Additionally, failure to detect LN metastasis status before treatment poses a high risk of recurrence post-surgery 5. This calls for an urgent need to devise an assistant method to establish LN status in PDAC patients before clinicians can make individual decisions.

Initially, carbohydrate antigen 19-9 (CA19-9) was suggested to be associated with pancreatic cancer 6. High CA19-9 levels are among the risk factors applied to estimate treatment protocols, whereas carcinoembryonic antigen (CEA) is a potential second biomarker for PDAC prediction 7, 8. A recent study found that the preoperative CEA^+^/CA12-5^+^/CA19-9 ≥ 1000 U/mL was potentially associated with poor surgical outcomes 9. Besides, patients presented with LN metastasis have been reported to accept neoadjuvant therapy first before surgery 10. Contrast agent-enhanced CT is a frequently adopted examination method in clinics, which assists surgeons to estimate the preoperative stage of PDAC 11, 12. Although CT imaging remains irreplaceable in the evaluation of resectability, it is limited to detecting the status of LN metastasis 13. Tumor features depicted by CT images are simple, such as its shape, size, and contrast intensity to normal tissues. However, these methods cannot predict LN status, particularly due to the influence of survival and recurrence rates.

Radiomics, as a recently emerging technology, has provided plenty of information on medical images that can uncover hidden characteristics of diseases, which are unclearly observed with naked eyes 16. This technology has been explored in clinical oncology, for instance, carcinoma of the lung 17, breast 18, bladder 19, and colorectal 20, to advance and promote the management of cancer. Moreover, radiomics can extract large amounts of high-dimensional quantitative features from medical images, including computed tomography (CT), magnetic resonance imaging (MRI), and positron emission tomography (PET) 16, 21. Thus, clinicians can establish correlations between features and the diagnosis or prognosis of cancer.

Herein, we developed a radiomics prediction model to predict LN metastasis status in PDAC patients before making a treatment decision. In this respect, this work aimed to uncover and validate a noninvasive approach for promoting detection of LN status in PDAC patients, which is crucial to comprehensively understand disease stratification and guide more accurate personalized treatment potentially in the future.

## Materials and Methods

### Patients

The patients enrolled in this study were pathologically diagnosed with pancreatic cancer and treated without any other treatments except surgical resection and lymph node dissection at the Second Affiliated Hospital of Zhejiang University College of Medicine, (Zhejiang, China) between 2014 and 2017. Those with portal venous phase CT and clinical preoperative variables were retrospectively analyzed.

**Figure 2:**
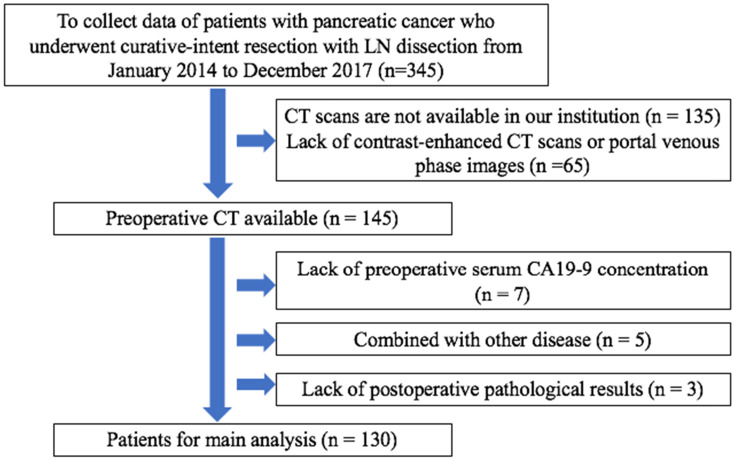
Selection criteria for patients. CA19-9: carbohydrate antigen 19-9.

Overall, we analyzed 130 patients who met the inclusion criteria (Figure 2), comprising 56 women (mean age, 65.1 years; age range, 59-71 years) and 74 men (mean age, 63.1 years; age range, 57-69 years). Patients were classified into the primary and validation cohorts based on surgery timing. In particular, 89 patients operated between January 2014 and December 2016 served as the primary cohort, whereas 41 patients diagnosed with PDAC between January 2017 and December 2017 served as the validation cohort. Approval for this retrospective study was issued by the Ethics Committee of The Second Affiliated Hospital Zhejiang University School of Medicine.

### Clinical Factors

Clinical variables, including, age, gender, CEA level, CA12-5 level, CA19-9 level 22, and CT examination dates were retrieved from the electronic medical record. Following logistic regression analysis, clinical factors with* P*-value < 0.05 as potential hazard factors were incorporated into the predicted model.

### CT Acquisition and Radiologic Evaluation

As part of regular treatment, all patients diagnosed with PDAC accepted a contrast-enhanced CT examination one week pre-operation. A multidetector CT (iCT 256, Philips, Netherlands) was adopted to acquire CT images with the following scanning parameters: 120 kVp; 300 mA, 80 × 0.6 mm collimation; a pitch of 0.8; a 512 × 512 matrix. Patients received an injection of 120 ml contrast material (Yangtze River Pharmaceutical Group, Jiang Su, China) via cubital vein at a rate of 3.0 - 4.0 mL/s before scanning. Afterward, we obtained images of the arterial phase and venous phase with delays of 30-35 seconds and 60-70 seconds, respectively. The reconstruction parameters included 3 mm slice thickness and 3 mm interval for contrast-enhanced images. Moreover, 2 practical and experienced radiologists reviewed all CT images to assess the features of each PDAC, including (a) tumor size, considering the maximum diameter on the transverse section as final; (b) tumor site, expressed as the head, neck, body, and tail of the pancreas; (c) LN status 23, considered as positive when presented 10 mm at least in short-axis diameter or hyperattenuating in the portal venous phase. The 2 experienced radiologists had knowledge of the PDAC diagnosis. They were, however, blinded to other patient details.

### Region-of-Interest Segmentation and Analysis

Radiomic characteristic features were extracted from contrast-enhanced CT images at 3 mm thickness. Using ITK-SNAP (version 3.6.0), two radiologists with clinical experience independently outlined the regions of interest (ROI) manually and segmented them around the tumor lesions from the portal venous. Both radiologists had knowledge of the diagnosis of PDAC but were unaware of the treatment details. Subsequently, features extracted from each segmented ROI and that could be classified as textural and non-textural features were analyzed using in-house software written in Python (version: stable; http://github.com/Radiomics/pyradiomics) 24.

Twenty images randomly selected were evaluated for the inter-observer reproducibility of the radiomics features. All images were assessed by a radiologist with at least 15 years (reader 1) experience in abdominal CT and a hepatobiliary surgeon with 5 years (reader 2) experience in hepatobiliary surgery and imaging. To evaluate the intra-observer reliability, reader 1 repeated the above procedure twice for 2 weeks. The intra-class correlation coefficient (ICC) values were counted to establish the stability of features for exclusion with ICC value < 0.75. The least absolute shrinkage and selection operator (LASSO) regularization were applied to verify the penalty coefficient with 10-fold cross-validation. This allowed us to choose the optimal features from the primary cohort.

### Developing a Radiomics-based Model

A radiomics prediction model was constructed through multivariable logistic regression analysis from the primary set. Then, we executed the variance inflation factor (VIF) to establish whether severe collinearity existed in variables. LASSO algorithm analysis was performed to explore the prediction model, which was then validated for predictive effectiveness in the validation cohort. Also, we constructed a radiomics prediction model, integrated with the clinical data selected via multivariate logistic regression analysis with radiomics features. Eventually, a radiomics nomogram was constructed as an auxiliary means for clinical use.

### Performance and Validation of Prediction Model

The ROC curve 7 and AUC 24 were adopted to quantify the predictive and diagnosis potential of established models 25. Comparison of various AUCs among different models was achieved using the DeLong algorithm 26. We also applied the calibration curves to evaluate the predictive accuracy of the radiomics prediction model, via the Hosmer-Lemeshow test. An independent dataset was applied to examine the internal validation of the radiomics-based model.

### Clinical Use

The clinical values were evaluated via decision curve analysis (DCA), whereby we calculated the net benefits of different threshold probabilities in radiomics, clinical, and CT models.

### Statistical Analysis

Continuous variables were presented as mean ± standard (SD), whereas categorical variables were analyzed via the χ^2^ test or Fisher's test. ROC curves, AUC, nomograms, calibration, and decision curves were generated using the R package (version 3.6.1). For all other statistical data analyses, SPSS version 21.0 (IBM) was applied. All statistical tests were 2-sided. *P* < 0.05 denoted statistical significance.

## Results

### Patient Characteristics

Patients diagnosed with PDAC in our hospital between January 2014 and January 2017, were enrolled in this study. The characteristics of patients showed no differences between the two sets (Table 1). The rate of LN metastasis was 50.1% (45 of 89) and 41.5% (17 of 41) in the primary and validation cohorts, respectively. However, we found no differences between the two cohorts (*P* > 0.05). Based on the CT report, 6 patients (22.2%; 6 of 27) without LN metastasis were under-staged, whereas 47 patients (45.6%; 47 of 89) with LN metastasis were over-staged.

### Radiomics Features Selection and Radscore Calculation

**Figure 3:**
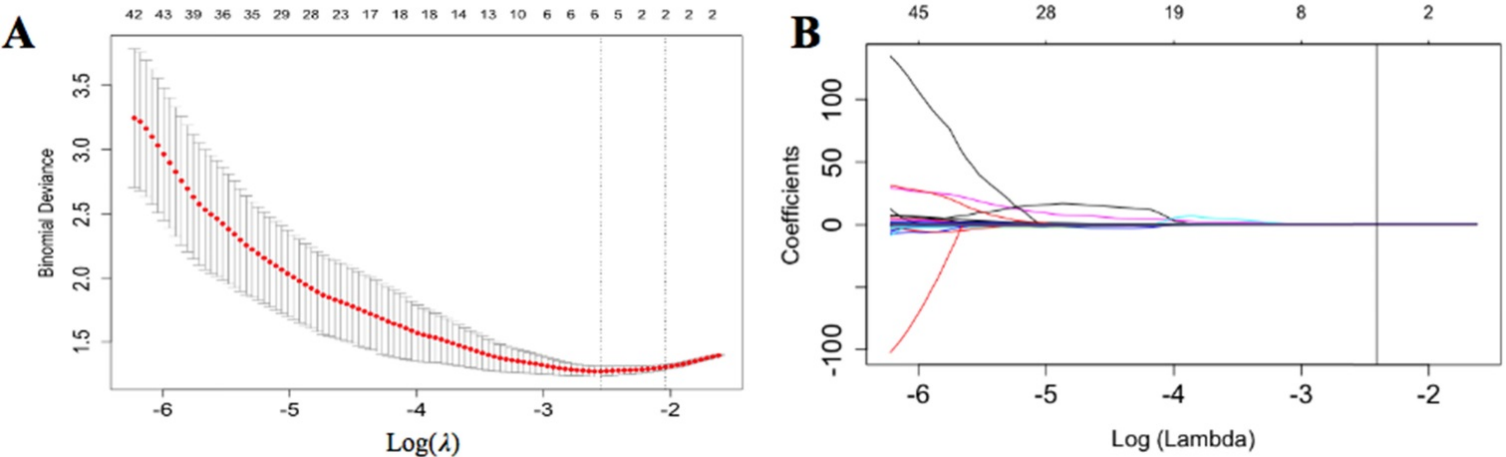
** Radiomics features selection.** (**A**) The tuning parameter (λ) selected in the LASSO algorithm through 10-fold cross-validation. The binomial deviance was drawn versus log (λ). Two vertical lines represent the optimum values using the minimum criteria and 1 standard error of the minimum criteria (1-SE criteria). The optimal λ value of 0.067 was picked. (**B**) LASSO coefficient profiles of 116 radiomics features. A vertical line was drawn at the value selected using 10-fold cross-validation, where optimal λ generated four coefficients.

We selected approximately 116 stable features for further analysis (Figure 3). According to the LASSO logistic regression model, 5 LN metastasis-related radiomics features with coefficients were selected in the primary cohort. They comprised three shape-related features, a gray-level co-occurrence matrix (GLCM) feature and a gray-level size zone matrix (GLSZM). After that, we computed a radiomics score (named radscore) as the radiomics signature using the formula:

Radscore = -1.90+1.84e-04*original_glcm_LargeDependenceLowGrayLevelEmphasis+3.18e-06*original_glszm_LargeAreaLowGrayLevelEmphasis+3.16e-02*original_shape_Maximum2DDiameterRow+1.75e-02*original_shape_Maximum3DDiameter+0.35*original_shape_MeshVolume. Where *glcm* denotes a gray-level co-occurrence matrix;* glszm* denotes agray-level size zone matrix, including features that describe the distribution of size and grayscale; the s*hape* represents the tumor size and shape of the ROI. The radscore of each patient was recorded for analysis

### Diagnostic Validation of Radiomic Signature

There were different radscores in predicting LN metastasis between the primary and validation cohorts. The radscore of patients with or without LN metastasis was -0.23 and 0.14 (median, *P* < 0.01) and -0.0006 and 0.77 (median, *P* < 0.01) in the primary cohort and validation cohort, respectively Additionally, the value of AUC of radscore was 0.77 (95% CI: 0.68, 0.87) and 0.76 (95% CI: 0.61, 0.91) in the primary cohort and validation cohort, respectively; this implied a good prediction effect.

### Development and Validation Prediction Models

**Figure 4:**
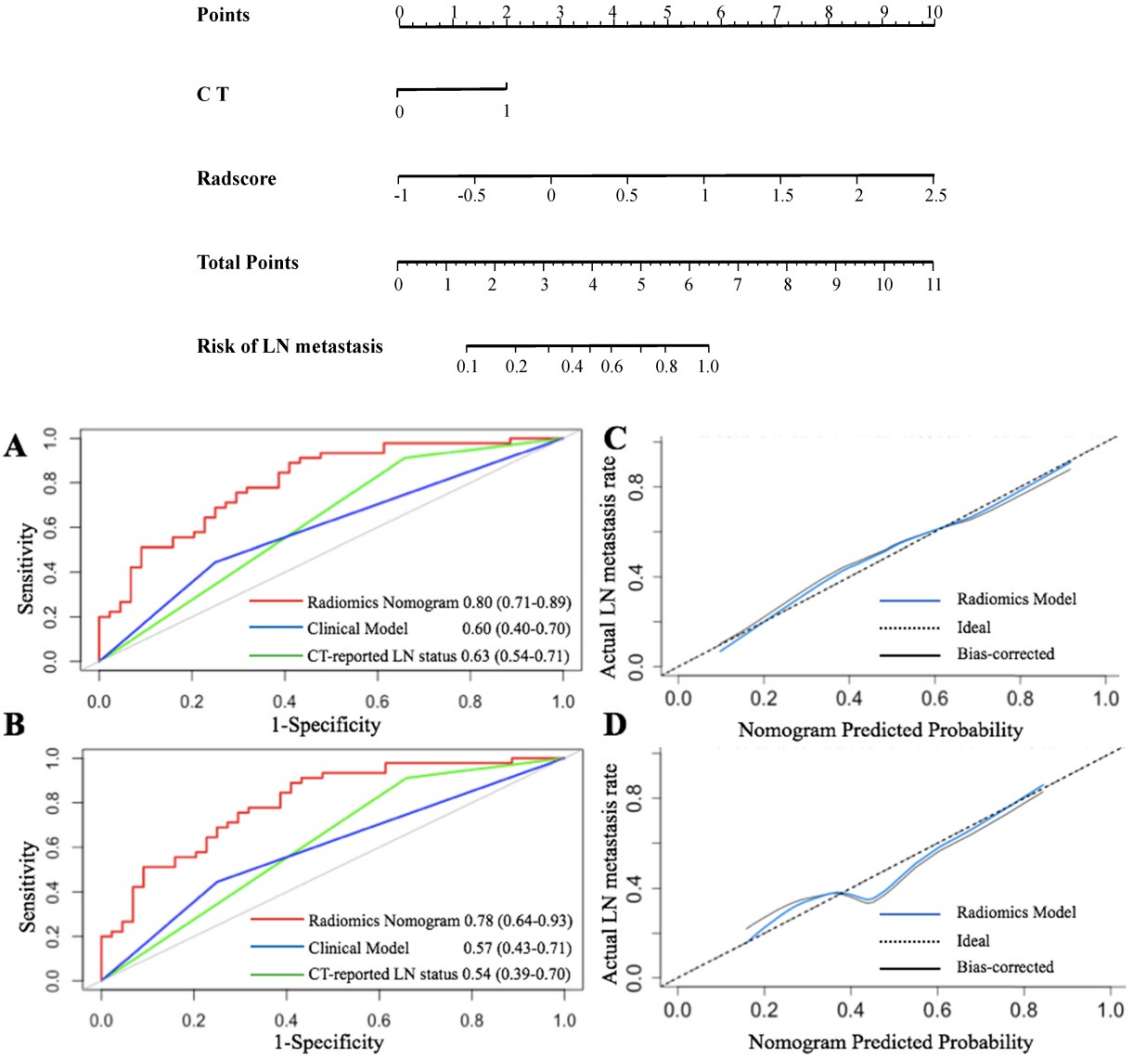
** Radiomics nomogram for predicting LN status accompanied by ROC and calibration curves.** A radiomics nomogram was established based on the primary cohort, with the incorporation of radiomics features and CT only. (**A** and **B**) represent the ROC curves among the radiomics, clinical, and CT score for predicting LN status in two cohorts, respectively. Calibration curves of the radiomics nomogram in the primary (**C**) and validation (**D**) cohorts.

The VIF of the alternative factors ranged from 1.04 to 1.67, which demonstrated no multicollinearity. A radiomics nomogram, integrating radiomics scores and CT-reported status as two predicted factors was constructed. Further, we developed a clinical prediction model utilizing two independent factors (CT-reported LN status and CEA^+^/CA12-5^+^/CA19-9 level) without a radiomics signature (Figure 4).

The ROC 7 curves are presented in Figure 4. The radiomics nomogram demonstrated the highest discrimination in the primary cohort (Figure 4A) [AUC value of 0.80 (95% CI: 0.71, 0.89)] compared to that of CT-reported LN status [AUC value of 0.63 (95% CI: 0.54, 0.71,* P* < 0.001)]. Besides, the clinical prediction model with an AUC of 0.59 (95% CI: 0.40, 0.69) showed poor performance than that of the radiomics model (*P* < 0.01).

Similarly, the radiomics nomogram exhibited the highest AUC (0.78, 95% CI: 0.64, 0.93) in the validation cohort (Figure 4B). This implied that the radiomics signature demonstrated a higher prediction quality compared to the clinical model (0.54, 95% CI: 0.39, 0.70, *P* < 0.05) or CT-reported LN status model (0.57, 95% CI: 0.43, 0.71, *P* < 0.01).

The Hosmer-Lemeshow test was further applied to generate the calibration curve and gave a *P* value of 0.90 in the primary cohort. This reflected a robust agreement between predicted and observed LN status (Figure 4C). However, the validation cohort was used to obtain a better agreement (Figure 4D). Besides, the *P*-value of the Hosmer-Lemeshow test in the validation cohort was 0.82, which depicted a good performance of the radiomics model in both cohorts.

After obtaining risk scores from the radiomics prediction model, we selected the optimal cutoff value of 0.045 (according to a total of 42 points) from the entire cohort based on the maximized Youden Index (sensitivity + specificity - 1). Generally, all patients were classified into low-risk and high-risk groups (*P* < 0.001). According to the risk classification, the radiomics prediction model demonstrated a sensitivity of 71.0% and a specificity of 69.1% for the prediction of LN metastasis status. The positive predictive value (PPV) reached 66.7% and the negative predictive value (TPV) reached 71.9%.

### Clinical Use

**Figure 5:**
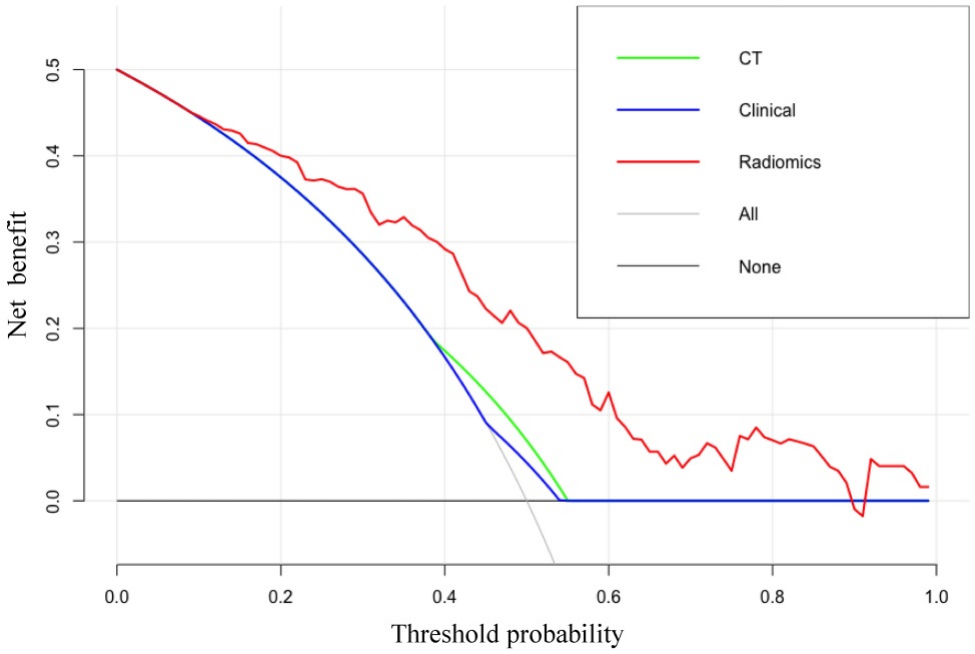
The DCA for each model. The y-axis indicates the net benefit. The red, blue and green line denotes the radiomics nomogram, the clinical and CT models, respectively. Gray denotes the “treat all” scheme and the black denotes the “treat none” scheme.

The DCA results for the radiomics-based model and CT-reported model are presented in Figure 5. The radiomics prediction nomogram achieved a significant benefit for the prediction of LN status than the “treat all” or “treat none” scheme and other methods for all threshold probabilities.

## Discussion

In this study, we developed a radiomics model, integrating four radiomics signatures and CT-reported LN status. It predicts the LN metastasis status in patients diagnosed with pancreatic cancer. Of note, the radiomics model differs from the traditional model as it integrates various aspects, such as CEA^+^/CA12-5^+^/CA19-9 level and CT-reported LN status, and elucidates apparent discriminative ability in both the primary (AUC, 0.80) and validation (AUC, 0.78) cohorts. This model is a promising noninvasive tool for individualized prediction of LN metastasis in PDAC patients before clinicians can make treatment decisions.

Compelling evidence implicates LN status as a vital factor for the determination of treatment options or the prediction of prognosis in pancreatic cancer patients 27. Herein, we revealed that the proportion of patients were misclassified according to clinical examination using CT images. Patients diagnosed with extensive lymph node metastasis, in most cases, accept neoadjuvant therapy shrinking tumor to benefit from surgery which improves their survival rates. Besides, discriminating malignant nodes from benign tumors based on metabolic activity still poses challenges due to small nodes and invisible features that potentially result in overtreatment or untimely treatment 28. Herein, the adoption of enhanced contrast CT to predict LN status was inaccurate and we advise that patients lined up for this procedure should not be treated via lymphadenectomy. Elsewhere, conclusions were based only on CT-scan by naked eyes which limited the full potential of precision medicine. Moreover, radiomics is crucial as it provides further and valuable information on clinical diagnosis. For instance, through multivariate logistic regression analysis of LN status metastasis in the primary cohort, we found that radscore and CT-reported LN status were associated with LN metastasis status (*P* < 0.05). The odds ratio demonstrated that both were risk factors, suggesting that the greater value was indicative of tumor metastasis. We then constructed a nomogram according to multivariate logistic regression analysis to evaluate and predict the probability of LN metastasis. This may assist clinicians to establish the LN status and make an individual decision on an appropriate treatment for patients. According to a recent study, the CA19-9 level may predict the prognosis of the disease 29. However, CA19-9 level or CT alone show poor performance than a radiomics nomogram which is highly promising in radiomics technology.

Despite these intriguing findings, this study has potential limitations. Although the radiomics nomogram demonstrates has immense potential to predict LN status, its diagnostic sensitivity and specificity are unsatisfactory for clinical application. Firstly, we only developed a radiomics nomogram using small sample size, and a single-center, this would limit its application. A large amount of dataset is, therefore, warranted to validate the accuracy and stability of our radiomics model to improve its practicability and stability. Secondly, we did not consider the tumor primary site, yet it may influence the clinical decision. Thirdly, we can infer from radiomics features that as the tumor appears more heterogeneous on radiomics analysis, there are higher chances of lymph node invasion. Also, after exploring the 3 shape-related features, we revealed that tumor size potentially affected LN metastasis status. However, tumor size and vascular invasion, taken as risk factors, showed unconsidered significance to LN which could be explained by the small samples and a single-center. Finally, we did not relate LN metastasis status or genotype identification to a specific pathological classification, which perhaps influences the assessment of disease procession for surgeons. Otherwise, deep learning-based methods, for example, convolutional neural network (CNN) can improve performance. With the CNN network, the LN status prediction can be discriminated by body part recognition, skipping several steps; this implies that one can integrate CT images into the model and output the result without the action by a doctor in the analysis 30, 31. However, the CNN model requires much more training data such that if we would collect enough data, CNN may be a better choice.

Collectively, the present study developed a noninvasive and convenient radiomics model to predict LN status before treatment decision. The model has a clinical application value for clinical staff to provide timely treatment and improve postoperative benefit in selected patients.

## Supplementary Material

Supplementary table S1.

## Figures and Tables

**Figure 1:**
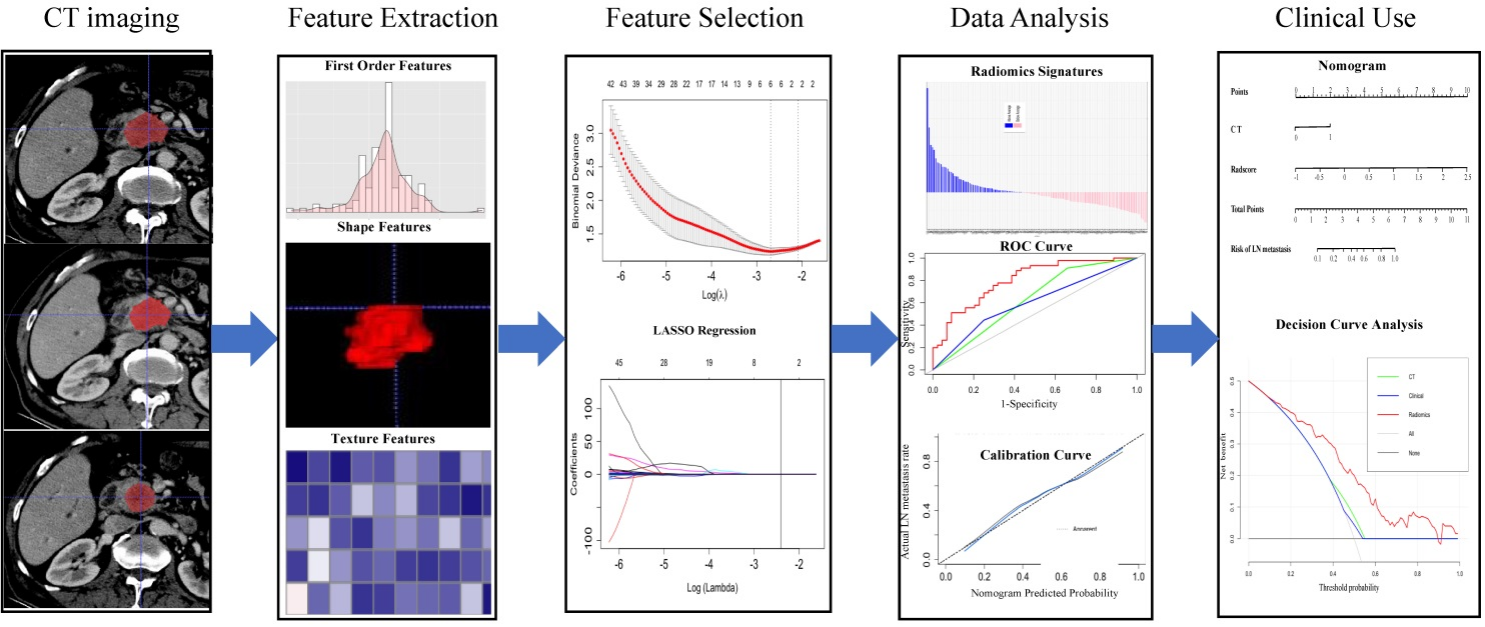
** The workflow of the essential procedures.** Original tumors are outlined on each axial portal venous phase CT slice. Amount of radiomics features were extracted from the region outline automatically to digitize tumor shape, intensity, and texture. Two essential steps were employed for feature selection. The radiomics model was established through a linear combination of selected features. The ROC and calibration curves were used to evaluate the efficiency of the radiomic model. Finally, the nomogram was constructed for individualized evaluation of clinical use.

**Table 1:** Characteristics of patients

Characteristic	Primary Cohort (n=89)			Validation Cohort (n=41)		
	Negative for LN Metastasis	Positive for LN Metastasis	P-Value	Negative for LN Metastasis	Positive for LN Metastasis	P-Value
**Age**			0.597			0.497
≤ 60 y	15 (53.6)	32 (52.5)		8 (66.7)	4 (33.3)	
> 60y	29 (47.5)	13 (46.4)		16 (55.2)	13 (44.8)	
Gender			0.063			0.853
Male	21 (38.9)	33 (61.1)		12 (60.0)	8 (40.0)	
Female	23 (65.7)	12 (34.3)		12 (57.1)	9 (42.9)	
**CA19-9**			0.054			0.577
≥ 1000U/ml	11 (35.5)	20 (64.5)		12 (63.2)	7 (36.8)	
< 1000U/ml	33 (56.9)	25 (43.1)		12 (54.5)	10 (45.5)	
**CEA**			0.057			0.680
≥ 5	7 (31.8)	15 (68.2)		10 (62.5)	6 (37.5)	
<5	37 (55.2)	30 (44.8)		14 (56.0)	11 (44.0)	
CA12-5			0.145			0.500
≥ 40	4 (30.8)	9 (69.2)		11 (64.7)	6 (35.3)	
<40	40 (52.6)	36 (47.4)		13 (54.2)	11 (45.8)	
**CT-reported LN status**			<0.05			0.292
	15 (78.9)	4 (21.1)		6 (75.5)	2 (25.5)	
	29 (41.4)	41 (58.6)		18 (54.5)	15 (45.5)	
**Tumor size (>3cm)**			<0.001			0.280
	24 (38.1)	39 (61.9)		18 (54.5)	15 (45.5)	
**Vascular invasion**						0.275
	9 (32.1)	19 (67.9)	<0.05	6 (46.2)	7 (53.8)	
Radscore*			<0.01			<0.01
	-0.23 (-0.45 to -0.04)	0.14 (-0.05 to 0.45)		-0.0006 (-0.31 to 0.27)	0.75 (0.01 to 1.01)	

Statistical analysis of patients with 95% CI; data in parentheses denotes percentages. CA19-9: carbohydrate antigen 19-9; CEA: carcinoembryonic antigen; CA12-5: carbohydrate antigen 12-5. *Data in parentheses shows interquartile range.

**Table 2:** Risk Factors for Lymph Node Metastasis in Pancreatic Cancer

Variable	Clinical Model		Radiomics Model	
	OR (95% CI)	*P*-Value	OR (95% CI)	*P-*Value
CA19-9	1.64 (0.97~1.05)	0.69	1.36 (0.58~3.19)	0.30
CEA	1.50 (0.65~3.47)	0.20	1.56 (0.59~4.12)	0.48
CA12-5	0.75 (0.30~1.87)	0.54	0.36 (0.12~1.09)	0.07
CT-reported LN Status	4.62 (1.65~12.93)	0.004	4.71 (1.48~15.03)	0.009
Tumor size	2.45 (1.49~4.03)	<0.001	2.12 (1.14~3.95)	0.894
Vascular invasion	1.16 (0.43~3.08)	0.768	1.10 (0.41~2.93)	0.852
Radscore	NA	NA	6.63 (0.80~54.69)	<0.001

Results of the multivariate regression analysis with 95% CI. NA = not available.
