# Supplementary material for: A radiomics model that predicts lymph node status in pancreatic cancer to guide clinical decision making: A retrospective study
Source: J Cancer. 2021 Aug 22;12(20):6050–7. doi: 10.7150/jca.61101 (PMC8425217; doi:10.7150/jca.61101)
Supplement: Supplementary file 1 — Supplementary table S1. [file jcav12p6050s1.pdf]

Supplementary File

Title: A radiomics model that predicts lymph node status in pancreatic cancer to guide clinical decision making: A retrospective study

Authors: Xiaoyuan Liang, Wei Cai, Xingyu Liu, Ming Jin, Lingxiang Ruan and Sheng Yan

Table 1 radiomic features extracted， detail description for each feature refer http://github.com/Radiomics/pyradiomics

Feature category	Feature list
firstorder	10Percentile、90Percentile、Energy、Entropy、InterquartileRange、Kurtosis、Maximum、Mean、MeanAbsoluteDeviation、Median、Minimum、Range、RobustMeanAbsoluteDeviation、RootMeanSquared、Skewness、TotalEnergy、Uniformity、Variance、log-sigma-1-0_Mean、log-sigma-1-0_Maximum、log-sigma-1-0_Median、log-sigma-1-0_Minimum、log-sigma-1-0_Energy、log-sigma-1-0_Entropy、log-sigma-1-0_Kurtosis、log-sigma-1-0_MeanAbsoluteDeviation、log-sigma-1-0_Skewness、log-sigma-1-0_Uniformity
Shape	Flatness、LeastAxisLength、MajorAxisLength、Maximum2DDiameterColumn、Maximum2DDiameterRow、Maximum2DDiameterSlice、Maximum3DDiameter、MeshVolume、MinorAxisLength、Sphericity、SurfaceArea、SurfaceVolumeRatio、VoxelVolume
glcm	glcm_Autocorrelation、glcm_ClusterProminence、glcm_ClusterShade、glcm_ClusterTendency、glcm_Contrast、glcm_Correlation、glcm_DifferenceAverage、glcm_DifferenceEntropy、glcm_DifferenceVariance、glcm_Id、glcm_Idm、glcm_Idmn、glcm_Idn、glcm_Imc1、

	glcm_Imc2、glcm_InverseVariance、glcm_JointAverage、glcm_JointEnergy、glcm_JointEntropy、glcm_MCC、glcm_MaximumProbability、glcm_SumAverage、glcm_SumEntropy、glcm_SumSquares
gldm	gldm_DependenceEntropy、gldm_DependenceNonUniformity、gldm_DependenceNonUniformityNormalized、gldm_DependenceVariance、gldm_GrayLevelNonUniformity、gldm_GrayLevelVariance、gldm_HighGrayLevelEmphasis、gldm_LargeDependenceEmphasis、gldm_LargeDependenceHighGrayLevelEmphasis、gldm_LargeDependenceLowGrayLevelEmphasis、gldm_LowGrayLevelEmphasis、gldm_SmallDependenceEmphasis、gldm_SmallDependenceHighGrayLevelEmphasis、gldm_SmallDependenceLowGrayLevelEmphasis
glrlm	glrlm_GrayLevelNonUniformity、glrlm_GrayLevelNonUniformityNormalized、glrlm_GrayLevelVariance、glrlm_HighGrayLevelRunEmphasis、glrlm_LongRunEmphasis、glrlm_LongRunHighGrayLevelEmphasis、glrlm_LongRunLowGrayLevelEmphasis、glrlm_LowGrayLevelRunEmphasis、glrlm_RunEntropy、glrlm_RunLengthNonUniformity、glrlm_RunLengthNonUniformityNormalized、glrlm_RunPercentage、glrlm_RunVariance、glrlm_ShortRunEmphasis、glrlm_ShortRunHighGrayLevelEmphasis、glrlm_ShortRunLowGrayLevelEmphasis
glszm	glszm_GrayLevelNonUniformity、

	glszm_GrayLevelNonUniformityNormalized、glszm_GrayLevelVariance、glszm_HighGrayLevelZoneEmphasis、glszm_LargeAreaEmphasis、glszm_LargeAreaHighGrayLevelEmphasis、glszm_LargeAreaLowGrayLevelEmphasis、glszm_LowGrayLevelZoneEmphasis、glszm_SizeZoneNonUniformity、glszm_SizeZoneNonUniformityNormalized、glszm_SmallAreaEmphasis、glszm_SmallAreaHighGrayLevelEmphasis、glszm_SmallAreaLowGrayLevelEmphasis、glszm_ZoneEntropy、glszm_ZonePercentage、glszm_ZoneVariance
ngtdm	ngtdm_Busyness、ngtdm_Coarseness、ngtdm_Complexity、ngtdm_Contrast、ngtdm_Strength
